# Supplementary material for: Are carbon emissions trading and green financial instruments synergistic? -Comprehensive quantitative research based on content analysis
Source: PLoS One. 2024 Mar 7;19(3):e0298601. doi: 10.1371/journal.pone.0298601 (PMC10919601; doi:10.1371/journal.pone.0298601)
Supplement: S1 Data — (ZIP) [file pone.0298601.s001.zip › Data/地级市Z_F工作报告.zip!/╕ú╜¿/╒─╓▌╩╨/└┤╫╘╣1⁄2╓┌║┼í╛┬φ┐╦╩2╛▌═°í┐_2006.docx]

各位代表：

现在，我代表市人民政府向大会作工作报告，请各位代表连同《漳州市国民经济和社会发展第十一个五年规划纲要（草案）》一并审议，并请政协委员和列席人员提出意见。

一、2005年和过去五年的工作回顾

过去的一年，在市委的正确领导下，全市上下坚持以邓小平理论和“三个代表”重要思想为指导，牢固树立和落实科学发展观，深入贯彻党的十六届中央全会、七届省委全会和八届市委全会精神，主动融入海峡西岸经济区建设，大力实施工业立市战略，同心同德，开拓奋进，全面完成市第十三届人民代表大会第六次会议确定的各项目标任务。实现地区生产总值626.36亿元，增长11%。其中一产增加值153.93亿元，增长3.7%；二产增加值249.86亿元，增长17.6%；三产增加值222.57亿元，增长9.3%。财政总收入50.61亿元，增长27.5%，地方级财政收入26.96亿元，增长23.6%。全市经济社会发展展现了良好趋势的新开端。

工业立市成效显现。工业总产值1002.2亿元（老口径），增长21.3%，工业产值含税率提升0.8个百分点；规模工业产值619.4亿元，增长26.7%；规模工业企业1195家，净增194家；食品、机械、材料、能源等四个主导产业和电子、家具、药业等三个重点产业产值占规模工业产值比重达84%。工业投资完成77.38亿元，增长49.2%，增幅比上年提高41个百分点；投资500万元以上工业项目680个，已投产项目308个。工业区整合取得新效益，角美（含龙池）开发区财税收入突破4亿元，招银、金峰等开发区财税收入超2亿元；各地工业园区引进了一批较大规模工业项目，获准4.2万亩建设用地中工业用地3.3万亩，建成通用厂房83.3万平方米。临港工业集中区初具规模，港口吞吐量突破2000万吨，增长37%。工业增长对地区生产总值、固定资产投资和财政总收入增长的贡献率分别达到54.8%、79.4%和60.4%。

农业农村稳定发展。农林牧渔业总产值269.01亿元，增长4.6%。新增粮食、水产、水果、花卉、林竹等基地27个（片），新增县级以上农产品生产加工龙头企业80家，新增无公害农产品生产面积10万亩，新增“绿标”产品18个。成功举办台湾名优水果展示展销会、荔枝交易订货会、?溪蜜柚节、海峡两岸茶文化节。启动建设台湾农民创业园，新引进漳台农业合作项目43个、新品种14个、新技术14项。税费改革成果得到巩固，集体林权制度改革基本完成确权发证任务，农信社改革进展顺利。全面选配村级农技员；深化贫困村、老区村、老革命基点村挂钩帮扶工作，市拨出5598万元帮助改善发展环境；培育一批新型农村合作组织，农业社会化服务体系进一步扩展。铺设农村水泥路1061公里，完成年度计划101%；水利“六千”工程完成年计划的125.5%。

强力实施项目带动。完成全社会固定资产投资195.22亿元，增长20.0%，增幅为1999年以来最高。110个省市重点项目完成投资99.8亿元，占固定资产投资51.1%；生产性项目完成投资68.8亿元，占重点项目投资68.9%。努力推进项目工作的深化，建立健全了项目生成、协调和督查机制，一批重大项目工程建设或前期工作取得新进展，万利达电子产品基地、灿坤家电产业基地、凯西钢铁、统一马口铁扩建、中集集装箱技改等一批大型项目竣工投产，招银港区1#、2#10万吨级泊位等大吨位码头建成投用，厦深铁路和龙厦铁路漳州段被列为今年开工建设项目；厦漳跨海大桥正式签约，华阳电厂三期工程列入国家发改委电力建设计划。

外向经济继续扩展。坚持以引台为主，扩大与港澳、海外经贸合作，对接珠三角和厦门、泉州等地产业延伸，全市经济外向度进一步提升。合同利用外资6.97亿美元，验资到资3.10亿美元，分别增长23.7%和23.9%；外贸出口25.98亿美元，增长20.5%；引进台资合作项目146项，合同台资2.57亿美元，凯景二期、统一二期、长春化工等一批较大规模新项目落地投建、投产。“4·9”漳台经贸恳谈会和第七届海峡两岸花博会暨农洽会合同外资分别达到3.65亿美元和1.78亿美元；东莞项目推介会现场签约项目44个，总投资5.25亿美元；“6·18”项目成果交易会对接项目和“9·8”投洽会合同项目总投资分别为30亿元和13.2亿美元，均创历史新高。

有效提升城市品位。市区新增建成区面积3.8平方公里。积极试行城市管理综合执法体制，进一步理顺市、区两级管理职责。把创建国家级卫生城市、环保模范城市、园林城市、文明城市活动有机结合，组织环境卫生、交通管理、市场管理、内河水系、街区景观等专项整治，以卫生全日保洁、车辆定位停放、餐饮定点设摊、拆除违章建筑、主街缆线落地等措施改善城市管理。华阳体育馆完成主体，江滨路完成路基，西洋坪大桥完成桩基，新增市区道路21公里，新增绿地96公顷，浦头港整治基本完成，三湘江整治全面启动。园林城市通过国家级验收，卫生城市、环保模范城市通过省级验收。各县（市、区）加大力度改善城市环境，南靖、长泰和龙海、诏安分别通过省级卫生城市（县城）复检或初检。

努力改善群众生活。城镇居民人均可支配收入11241元，增长11.1%，农民人均纯收入4690元，增长8.6%。城乡居民储蓄285.13亿元，比上年底增加14.5亿元。社会消费品零售总额增长11.8%。新增就业4.32万人，下岗人员再就业5188人，城镇登记失业率为4.3%，实现控制目标；转移农村富余劳力7.68万人，劳务派遣工作名列全省第一。2.5万名城市低保对象和8.6万名农村低保对象最低生活保障金全部及时足额发放；社会养老保险、医疗保险制度逐步健全并较好落实；残疾人等特困群体生活保障机制基本形成。清理拖欠工程款2.76亿元，其中政府项目清欠率达97.5%，农民工工资清欠率达98.98%，如期完成国务院和省政府下达指标。加强市场秩序整规工作，有效保持液化气正常供应。18个为民办实事项目全部完成。

积极发展社会事业。新增高新技术企业10家，高新技术产业产值150亿元，增长25%，增幅居全省第一；市级和11个县（市、区）全部通过全国科技进步考核。经济普查工作获得全国先进称号。巩固提升义务教育成果，中小学危房改造超额完成年度计划，芗城、龙海、长泰顺利通过“双高普九”验收，高中阶段教育入学率71.2%，比上年提高10个百分点，高考上线率居全省前列。市民族宗教局被国务院授予“全国民族团结进步模范集体”称号。成功举办闽南文化节，市木偶剧团获国际木偶节最高奖，市区香港路、台湾路街区获联合国亚太地区文化遗产奖，漳浦县获得全国文化先进县称号。朝阳医院一期工程完成主体，全市公共卫生疾控体系进一步形成，实行就诊初检信息共享等一系列减轻群众负担的措施，新型农村合作医疗试点工作在长泰顺利开展。成功举办市十运会；谢荔梅获得亚洲田径锦标赛女子三级跳远金牌，王猛健夺得全运会男子100米蛙泳冠军。芝山红楼被列入全国百家经典红色旅游景点。计生工作完成省委、省政府下达的责任指标，涌现出龙海、芗城和漳浦、华安等一批全国、全省计生工作先进县。连续七年保持耕地占补平衡，实施土地整理工程2.33万亩，第一期“青山挂白”治理通过省政府验收。组织九龙江水环境整治“零点行动”，市区饮用水源水质明显提升。创建“平安漳州”扎实开展，社会治安综合治理责任制省级考评位次前移，公安机关多项工作保持全国先进，重特大安全生产事故得到有效遏制。同时，人事编制、外事侨务、统计、审计、气象、地震、档案、方志、国防动员、民兵预备役、人防、老龄、妇女儿童、残联等工作也取得了新成效。

各位代表，2005年全市经济社会发展所取得的成绩，为我市“十五”计划的实施画上了圆满的句号。过去的五年成为我市经济社会发展最快最好、城乡面貌变化最大最新、人民群众受惠最多最实的五年。主要体现在：

发展思路趋于完善。2002年，市委、市政府确立了“工业立市、开放强市、实干兴市”的发展战略，在巩固农业优势的同时，集中力量发展工业经济。省委确定建设海峡西岸经济区之后，我市立足于对台、港口、农业优势，确定了打造两岸经贸合作基地、新兴制造业基地和优质食品供应基地，建设与厦门特区互动、对台合作紧密、对外开放领先的生态工贸港口城市的总体思路。同时，以发展工业经济为重点培育增长点，以发展民营经济和优化服务环境为重点着力突破点，以发展县域经济和增强财政实力为重点主攻薄弱点；按照“一区三片”的基本格局，开展比学赶超活动，推动县域经济梯度发展。去年初，根据市委建议，市人代会作出了坚持工业立市、建设生态工贸港口城市的决定，全市干部群众以这一总体思路凝心聚力，推进发展。去年以来，市委、市政府施行了依法实行税收优惠、合理把握工业地价、积极推行集约用地、行政管理“重在提请”、努力改善城市环境等一系列措施，使工业立市的总体思路不断丰富、完善和具体化。

综合实力显著增强。地区生产总值在五年间两次调整统计口径，全国经济普查核定去年为626.36亿元，五年增长65.8%，年均增长10.5%。三次产业结构比例由31.8∶32.5∶35.7调整为24.6∶39.9∶35.5。工业总产值、规模工业产值分别增长1.4和2.7倍，农业总产值增长24.5%，外贸出口增长2.6倍，固定资产投资增长51.7%，社会消费品零售总额增长60.4%，财政总收入增长1.2倍，其中税性比重提高8.05个百分点。尤其实施工业立市以来，与2002年基数比较，工业总产值、规模工业产值分别增长82.6%、156.2%，外贸出口增长233.7%；财政总收入增长99.4%，占地区生产总值比重提高2.04个百分点。

深化改革破解难题。龙溪轴承等一批国有企业实现股份化改制并顺利上市，国企整体质量和效益提升。出台一系列鼓励台资、外资投资兴业措施，五年实际利用外资35亿美元（历史可比口径）。突出引导、扶持民营经济加快发展，民营企业产值和投资总量分别增长1.8倍和2.1倍。市区供水和污水、垃圾处理系统进入产业化经营轨道。整合城市资产，获得国家开发银行授信26亿元用于市政建设。在全省率先建立行政服务中心，再造行政审批流程，提升服务效率。以积极措施缓解企业融资、用地、用电、用工等方面的难题，促进了工业经济持续快速增长。尤其近几年来，在抗击“非典”和连年发生的冻灾、旱灾、洪灾及频发的台风袭击中取得重大胜利，有效保障了经济运行和社会事业平稳发展。

发展环境不断优化。高新技术产值增长5倍；建立科技创新中心；龙轴、万利达、灿坤等一批企业具有较强的自主开发能力；生产主体与科研机构、大专院校的协作体系基本形成。义务教育向高水平、高质量推进，达标中学由14所增至35所，高中阶段毛入学率由32%提高为60.9%；以漳州职业技术学院为先导基本建成高校园区，支持漳州师范学院扩展规模和厦门大学漳州校区工程建设。引进各类科技人才580名。新增高速公路247公里、等级公路466公里，万吨以上码头泊位6个，被交通部确定为国家级运输枢纽城市。市区建成区面积由32平方公里扩大至43平方公里，城市化水平由34.9%提高到38.7%。先后荣获全国创建精神文明先进城市、全国科技先进城市、中国优秀旅游城市、国家园林城市和全国双拥模范城市三连冠等荣誉称号。

城乡群众受益良多。城镇居民人均可支配收入从7059元增至11241元，农民人均纯收入从3530元增至4690元。城乡居民储蓄由135.68亿增至285.13亿元。全面取消农业税、特产税、农村屠宰税和统筹提留，减轻农民负担近10亿元；支持农户生产贷款16.8亿元；转移农村富余劳力36.4万人；建设造福工程和灾民安置房近70万平方米；改造农村中小学危房46.6万平方米；71%行政村实施卫生饮用水工程；新修农村等级公路4000多公里。新增城镇就业人数16.5万个，下岗再就业达60%以上，城镇登记失业率控制在4.12%；建成城镇住宅441.9万平方米，城镇居民人均住房面积增加11平方米，建成经济适用房25.3万平方米；完成市医院门诊大楼等一批医疗、文化、体育设施；市区人均公共绿地面积由6.32平方米增至11.95平方米；实施食品放心工程取得阶段性成效，初步建立消费者权益保护机制；社会保险、社会救助、社会福利、优抚安置相结合的社会保障体系初步形成。城乡社会安定，群众对社会治安满意率年年超90%。

过去的五年，漳州各项事业取得长足进步，但仍然有一些突出问题。主要有：经济总量较小，综合实力较弱，发展速度较慢，科技含量较低，与兄弟地区比有较大差距；产业集中度较低，市场体系较弱，产业与市场互动共荣效应不足；农村基层基础仍较薄弱，农业抗灾能力仍较脆弱，农业产业化及科技水平有待进一步提高；临港工业开发区基础设施建设滞后，吸引大型骨干项目能力较弱，经济发展缺乏支撑性、后续性大项目；地区之间、城乡之间发展不平衡，公共财政总体实力偏弱，县乡财政仍较困难；城乡群众收入增长较为缓慢，就业再就业压力较大；社会事业发展相对滞后，中心城市规划、建设和管理存在薄弱环节，县城集镇发展滞缓，社会治安、市场秩序、安全管理等方面存在一些问题；官僚主义、形式主义、腐败现象仍然存在，行政服务环境有待进一步改善。这些问题，有前进中的困难，也有体制上的弱节、政府工作上的缺失，将在改革发展中逐步化解，促进全市各项事业迈上新台阶。

各位代表，过去五年我市的成就与辉煌，是在市委的正确领导下，在各级人大及各位代表的依法监督和有力支持下，在各级政协及各位委员的民主监督和积极参与下，全市人民齐心协力、艰苦奋斗取得的。每一个进步，都凝聚着全市广大干部群众的心血、智慧和汗水。在此，我代表市政府，向积极投身漳州改革、建设与发展的广大干部群众，向各位人大代表、政协委员，向各民主党派、人民团体、社会各界人士，向大力支持我市各项事业发展的省、部属各单位、驻漳部队和武警官兵，向长期关心支持漳州建设的港澳台同胞、广大侨胞表示崇高的敬意和衷心的感谢！

二、“十一五”时期奋斗目标和总体要求

已经开启的新的五年，是我市全力打造生态工贸港口城市、建设海峡西岸经济区重要增长极的历史机遇期，也是加速推进全面建设小康社会的关键时期。根据《中共漳州市委关于制定国民经济和社会发展第十一个五年规划的建议》，市政府组织编制了《漳州市国民经济和社会发展第十一个五年规划纲要（草案）》（以下简称《纲要》），提请这次大会审议。

《纲要》提出“十一五”我市经济社会发展的总体要求：高举邓小平理论和“三个代表”重要思想伟大旗帜，认真贯彻党的十六届五中全会和省委七届十次全会精神，坚持以科学发展观统领经济社会发展全局，全面落实省委建设海峡西岸经济区的战略部署，紧紧围绕持续发展、科学发展、创新发展、为民发展，继续推进工业立市和项目带动战略，把区位优势转化为发展优势，把农业优势转化为工业发展优势，把资源优势转化为经济优势，全力打造两岸经贸合作基地、新兴制造业基地和优质食品供应基地，努力将漳州建设成为海峡西岸生态工贸港口城市和区域经济发展的新增长极，为提前实现全面建设小康社会、率先基本实现现代化而努力奋斗。

《纲要》提出了我市“十一五”期间经济社会发展的基本目标：在优化结构、提高效率和降低消耗的基础上，实现地区生产总值年均增长10%，力争更快更好些；2007年地区生产总值和人均地区生产总值比2000年翻一番，2010年地区生产总值超过1000亿元，三次产业结构调整优化为18∶44∶38；财政总收入超过110亿元，城镇居民人均可支配收入年增长7%以上，农民人均纯收入年增长6%以上；人口自然增长率控制在7‰以内，城镇登记失业率控制在4%以下；资源利用效率显著提高；经济竞争力和综合实力显著增强，在海峡西岸经济区的地位和作用更加凸显。

为实现上述目标，各级政府必须按照省委、市委的要求，把握好以下几个方面：

一、准确定位。当前，全球经济周期性上升，国内市场经济体系进一步拓展。党中央关于支持海峡西岸经济发展的重大决策，为我市带来了新的发展机遇。我市面临台湾海峡，毗邻厦门特区和珠江三角洲等发达地区，具备港口、腹地、电力、水源、劳力、气候等资源相对集中的比较优势，必须把握好历史机遇期，在建设海峡西岸经济区总体格局中，准确定位，保持清醒，创新思路，有所作为。要坚持工业立市发展战略，继续打造两岸经贸合作基地、新兴制造业基地和优质食品供应基地，努力构建生态工贸港口城市；要以培育港口经济为重点，加快港口岸线资源综合开发和合理利用，依托临港工业集中区，引进大型工业企业，壮大机械、电子等主导产业，培育石化、钢铁等新兴产业，大力发展港口物流业；要在我市发展总体构想的框架下，不断充实、调整、丰富、完善基本思路和具体举措，推动我市在海峡西岸经济区建设中展示新作为，做出新贡献。

二、主动融入。要立足于我市与台湾关系密切、台商投资密集的独特优势，按照省委关于依托“五缘”、拓展“六求”的要求，不断开拓漳台经贸科技文化交流的渠道和方式，力求在引进台湾大中型项目、高新技术项目上取得突破，力争建设台商投资区。要立足于我市与珠江三角地区接壤的区位条件，主动对接珠三角产业扩展，联络骨干企业引台引外，发展产业配套项目。要立足于厦门湾岸线资源整合的有利时机，主动参与厦门湾区域经济的产业培育和分工，积极参与构建厦漳泉城市联盟。要立足于我市区域经济加快发展的良好态势，继续按照梯度推进的发展步骤，实施“加快开发两湾、延伸做强两翼、拓展繁荣两线、联动发展四区”的发展布局，努力使我市成为海峡西岸经济发展的重要增长极。

三、拓展优势。一方面，要继续巩固、发展我市区位、资源和农业等方面的传统优势，把它们提升为发展优势；另一方面，要结合我市的市情和发展取向，努力构建新的优势。要在打造气势上构建新优势。尽管与先进地区比我市有一定差距，但我市更具优势、潜力和空间，并已形成强势发展的新开端，我们要自尊、自信、自强，营造加快发展的新气势。要在项目带动上构建新优势。必须强化抓发展就要抓项目、抓项目就是抓发展的理念，进一步健全项目的生成、引进、协调、服务、落实机制，以把项目紧紧抓在手中来实现发展措施到位。要在优化服务上构建新优势。各级行政机关应以服务发展、服务基层、服务企业、服务群众为己任，倡导“重在提请”的管理理念，推行“和谐互动”的管理方式，努力提高服务水平和质量。要在落实责任上构建新优势。必须坚决落实每个项目、每个任务、每个时段的责任目标，创造既积极有为又确保落实的发展成果。

四、持续发展。推进漳州更快更好地发展，是漳州百姓的热切期盼。要持之以恒地坚持发展第一要务，巩固、扩展近几年来我市已形成的快速发展的良好势头，把正在进行的有为、有效、有益的事业扎扎实实地推向新的发展阶段。工业优先发展要继续凸显。至2010年，规模工业产值超1400亿元，占全部工业产值85%以上；形成年产值超百亿产业集群3—4个、年产值超50亿工业企业3—5家，年纳税超亿元企业8—10家。农业基础优势要继续强化。农业增加值超180亿元，农产品加工率达65%以上，特色农业、有机农业、品牌农业的基地化生产和组织化经营形成体系，提升农业的产业化、机械化、商品化、无害化、外向化程度。服务产业发展要继续加快。服务业增加值超380亿元，年均增长11.3%，商品市场、专业市场、物流市场体系基本健全，与工农业产业互动作用显著增强。公共服务功能要继续提升。厦深铁路、龙厦铁路漳州段建成投运，争取投建港尾、古雷、东山、诏安支线；增强厦门湾漳州港区国内、国际航运综合输运能力和物流配送能力，建设以古雷港为重点的东山湾港区深水码头，港口吞吐量超4000万吨；建设高速公路复线；整合、优化宽带通信网、数字电视网和新一代互联网基础设施；完善防灾抗灾指挥信息化网络和基础工程体系。城市辐射能力要继续增强。中心市区建成区超50平方公里，人口超50万人；市区东区基本建成，行政服务及商贸、科技、教育、文化等功能基本健全；梯度推进九龙江两岸东进西扩开发建设，以水仙大街为主轴构建沿江新景观；提高老城区改造管理标准，优化人居环境；加强县城和中心集镇规划建设管理，全市城市化水平达48%。社会事业发展要继续增效。科技创新能力和对经济成长贡献率明显提升；提高九年义务教育质量，高中阶段教育毛入学率达80%以上，职业教育、高等教育、成人教育、幼儿教育、特殊教育全面发展；具有地方特色的群众性文化生活丰富多彩；城乡公共卫生和医疗服务体系基本健全；群众性健身活动全面开展；人口、资源、环境三项基本国策有效落实；节约型、环境友好型社会建设全面启动；多层次社会保障体制进一步完善；“信用漳州”、“平安漳州”建设取得新成果。

三、2006年政府工作主要任务

今年是实施“十一五”规划的开局之年,也是全力推进海峡西岸生态工贸港口城市、建设社会主义新农村的基础之年，做好今年工作意义十分重大。今年我市国民经济和社会发展主要预期目标是：地区生产总值增长10%以上；规模工业产值增长26%；农业生产总值增长4%；财政总收入增长20%，力争25%，其中地方级财政收入增长16%以上，税性比重进一步提高；全社会固定资产投资增长18%以上；外贸出口总值增长15%以上；实际利用外资增长10%以上；社会消费品零售总额增长11%以上；城镇居民人均可支配收入增长8%，农民人均纯收入增长6%以上；城镇登记失业率控制在4%以内；人口自然增长率控制在6.7‰以内。

实现今年各项目标任务，要重点抓好以下工作：

（一）培育工业产业集群，构建港口经济框架

发展临港产业。要在全省建设海洋强省规划指导下，加快编制海洋经济发展规划。着力培育厦门湾南岸、东山湾两个临港产业集聚区。加快招银港区7#-9#泊位、古雷港区一德石化、港尾海洋石化等项目建设；抓紧漳州开发区第四区建设前期工作，推进组合港建设，完善港口配套。以招银、角美、金峰、蓝田、古雷、常山等工业区为重点，大力推进临港工业园区建设，做好一批大型重化工业项目洽谈引进工作，争取临港产业开发取得实质性进展。积极推进招银、后石、古雷三个物流园区建设，逐步构建以码头体系、仓储输运、临港工业、物流产业相互配套、相互促进的港口经济集群。

推进产业聚集。以食品、机械、材料、能源等主导产业和电子、家具、药业等重点产业为基础，培育产业集群。主动对接厦门湾产业集群，对接周边地区市场体系，对接珠三角产业转移，提升优化传统产业，培育拓展新兴产业。积极参与全省重点产业集群建设，扶持厦漳视听产品、厦漳闽台合作农产品加工、漳州家具等3个省定重点产业集群延伸链条壮大规模。引导一批龙头企业建设营销、研发中心，促进关联企业加快集聚。积极筹建企业孵化中心、模具加工中心和生产技术人员培训基地，继续以项目带动标准厂房建设，增强产业公共服务功能。鼓励、支持企业培育品牌，带动产业扩展。

狠抓重点项目。加快600个投资500万元以上工业项目和290个年新增产值1000万元以上的工业经济支撑项目建设，抓好200个已签约项目的跟踪落实，完成工业项目投资100亿元以上。引导扶持大中企业特别是产值亿元以上企业增强自主创新能力，加快产能、效益有效扩张。抓好45个省级工业经济增长点的落实，从煤电油运及用地、用工等生产要素服务方面给予重点扶持，促进扩大规模，增强核心竞争力。

增强民营活力。完善《关于进一步推动民营经济发展的若干意见》，编制《中小企业发展规划》。以积极有效的措施激发民营企业的创业热情，扩展民营投资主体。引导建设一批民营工业项目，培育壮大一批民营工业企业，努力提高民营工业总量、质量和效益。着力完善民营经济服务体系，开展中小企业创业者培训，支持发展中小企业信用担保体系，加强行业公会建设，为民营经济发展搭建良好的服务平台。

（二）努力增加农民收入，积极推进新农村建设

深入贯彻落实中央一号文件精神，按照“生产发展、生活宽裕、乡风文明、村容整洁、管理民主”的要求，制定建设社会主义新农村总体规划和实施方案，统筹谋划建设新农村的目标、任务、步骤、政策和项目，实现新农村建设良好开局。

提升农业综合生产能力。稳定粮食生产总量。深入实施《绿色行动计划》，加快优质食品供应基地、对台农业合作基地和农产品出口基地建设。推行农业标准化生产，扩大无公害农产品、绿色食品、有机食品的总量和规模。引导企业实行“QS”认证，鼓励开展HACCP、ISO14000、SA8000等国际认证。扶持105家市级以上农业产业化龙头企业建基地、创品牌、拓市场。加快30个市级农民合作经济组织试点发展。提高农业科技创新和转化能力，大力开发节约资源和保护环境的农业技术，加快发展循环农业。

千方百计增加农民收入。全面落实各项支农政策，扶持促进农村二、三产业更快发展。着力发展高值水产、名优水果、时令蔬菜、食用菌、茶叶、花卉等一批主导特色产业，促进农业增效。继续抓好农村劳动力培训和劳务派遣工作，提高农民就业能力，转移农村劳力7.6万人以上，增加农民非农收入。完善农产品市场网络建设，扶持发展农产品营销合作组织，继续举办各种大宗特色产品展销活动。实行整村推进的扶贫开发方式，积极推广产业扶贫、小额信贷等有效办法，促进低收入农户脱贫致富。

加强农业保障体系建设。继续抓好“六千”水利工程、农村“年千公里”水泥路工程和“村村通”广播电视工程建设；重点抓好防汛抗旱工程建设和水毁工程修复，提高防灾减灾能力；进一步巩固、改造沿海防护林体系；继续推进水利化县、农村电气化县建设；扩大通讯、供电、信息等网络在农村的覆盖面。加强禽流感等重大动植物疫病防治，做好农、林、水病虫害防治和渔业生产安全、森林防火工作。

深化农村工作机制创新。认真实施第二次全国农业普查工作。巩固农村税费改革成果。充分发挥农村三级核心网络作用，完善干部下派驻村制度和市领导挂钩贫困村制度。推行“乡财县管乡用”的财政管理方式。实行市、县医院挂钩乡镇卫生院制度。深化集体林权制度、水利工程管理体制和小型水利设施产权制度改革，完善水域滩涂养殖证制度，在依法、自愿、有偿基础上扩大土地经营权流转。扩展创建信用村（镇）活动。建好村级农技员、国土资源和规划建设协管员、计划生育协管员、乡村医生、治安调解员等“五大员”队伍。落实农村“一事一议”，深化“民主示范村”创建活动，规范村务公开，发展集体经济，积极稳妥化解村级债务。

大力推进村容村貌整治。坚持规划先行、试点起步、稳步推进的原则，谋划好新农村建设。组织新村建设规划修编，认真抓好12个镇120个村等示范村镇整治工作。开展农村垃圾治理“家园清洁行动”，做到村道硬化、空地绿化、水沟净化，组织好治脏、治乱、治差和改水、改厕、改圈工作，推广农村户用沼气池，探索建立农村环境卫生管理长效机制。尊重村民意愿，加强规划引导，充分考虑群众承受能力，落实特困农户扶持办法，有计划推进新村规划建设改造。市、县（市、区）财政安排一定资金支持村容整治。

（三）推动投资持续增长，增强市场拉动实效

切实加强重点项目建设。今年计划安排重点项目245个，其中在建重点项目168个，年度投资118亿元，列入省重点项目22个，年度投资21.3亿元。重点推进灿坤家电、长春化工、后石电厂三期、万利达产业集群、漳州港7#、8#、9#泊位、江滨大道、山梅公路等一批在建重点项目建设，争取超额完成年度投资计划；积极推动漳州开发区四区多用途泊位、龙厦铁路和厦深铁路漳州段等63个预备重点项目开工建设；促进千鼎特殊钢、南山景区、九龙江防洪堤四期等14个重大前期工作项目从储备阶段转为实施阶段。坚持和落实项目分级管理、领导挂钩、责任到人、定期督查等制度，确保项目如期建成投产。

有效扩大市场消费需求。实施《漳州市中心城区商业网点规划》，加快完善粮食、钟表、家具、食品、汽车、茶叶等专业批发市场和吉马、聚善堂、沃尔玛、好又多等物流市场建设，提高新华都、芗客隆、国美、永乐等大型超市电子信息化水平。大力发展行业协会、专业协会、信息咨询等中介服务组织。培育房地产市场，促进住房消费。以漳台民俗史迹为主线，着力培育特色旅游品牌，整合精品线路，引资投建高星级宾馆，壮大旅游产业。组织开展贸洽会、定货会、新产品展示会等各种营销活动，鼓励和引导商业服务企业深入农村集镇发展连锁店、便利店、超市等流通业务。扩展本地产品营销网络，扩大社会消费。

不断优化金融服务功能。围绕增加投资、扩大内需和产业结构调整，进一步加强银政、银企沟通合作，搞好项目对接。鼓励金融机构加快业务创新，优化资金投向，加大对优势产业、重点项目、骨干企业和中小企业、民营经济的支持。做好城市资产整合、运营，争取金融资金投放向城市公共设施建设重点项目倾斜。以推进农村信用户、信用村建设，搞活农户小额信贷。加强金融监管，防范和化解金融风险。继续做好农村合作基金会、城信社后续清理工作。

（四）发挥漳台合作优势，提升对外开放水平

漳台合作要有新拓展。要认真落实省委关于依托“五缘”优势、拓展“六求”作为、加强对台交流十大窗口建设的要求，推动漳台经贸文化交流拓展内涵、提高层次。加大对台商投资密集区的招商力度，继续办好“4·9”漳台经贸恳谈会，把海峡两岸花卉博览会的内涵向经贸科技文化交流的全领域扩展，努力把漳州建成全国重要的两岸经贸合作基地。发挥漳州海峡两岸农业合作实验区先行优势，扩展台湾农民创业园规模和成效，使我市在海峡两岸（福建）农业合作实验区建设中充分发挥示范作用。全面落实《鼓励台商投资与扶持台资企业发展的若干意见》，在台资企业的准入、用工、用地、融资等方面实行实质性的扶持措施，推动漳台合作实现更大突破、更快发展。

外引内联要有新实效。按照精心策划、创新机制、搞好服务、提升水平的要求，积极组织参加“9·8”投洽会和“6·18”项目成果交易会等招商活动。以引进重特大项目为突破点，以提升外事、侨务、对台工作实效为纽带，瞄准跨国公司和台港澳大企业，做深做细招商项目前期工作，力争有一批重大支撑项目生成落地。坚持走出去对口招商，深入珠三角、长三角和厦门、泉州等发达地区举办招商推介会，寻求区域协作、产业对接。加强引资项目跟踪服务，切实提高签约项目的报批率、履约率和投产率。

外贸出口要有新进展。进一步完善领导挂钩重点出口企业服务制度，落实出口鼓励政策，指导企业有效规避国际贸易风险，支持企业做大出口总量。组织企业参加广交会、华交会、商交会，支持企业境外展销开拓国际市场。积极推动企业进行国际通行质量管理体系、环境管理体系和行业认证，鼓励企业创建名牌，力争今年申报商务部的出口品牌有所突破。建立健全“大通关”协调机制，切实提高出口通关、退税速度，促进我市出口持续增长。

（五）增强中心城市功能，推动区域经济发展

提升中心城市发展水平。继续按照“建设新区、开发沿江、改善旧城”的发展思路，扩展中心市区，优化城市形象。加强对250平方公里中心城市规划区的统一管理。加快城建重点项目建设，确保江滨大道、南昌路、龙文北路、金峰北路、丹霞路、八号路、零号路一期工程全线贯通；实现西洋坪大桥主体合拢；完成金峰污水处理厂主体工程；动工建设江滨大道西延伸段、瑞京路西段、钟法路南段；做好漳华路、延安南路、北环路、西洋坪大桥南连接线等工程全面启动的前期工作。抓紧规划沿江城建项目，带动南岸片区联动开发。加强城市环境整治与管理，深入开展创建国家卫生城市和环保模范城市活动，巩固国家园林城市创建成果，加紧浦头港、三湘江、九十九湾整治，继续实施绿化、美化、亮化工程，增配公共交通和环卫保洁设施，推行污水处理产业化运作。发挥城市综合执法局作用，构建长效管理机制，提高城市管理水平。

促进县域经济联动发展。全面落实支持县域经济发展的政策措施，鼓励、支持县（市）发展优势产业，加快推进工业化、城镇化进程。继续开展比学赶超活动，推进县域经济按“三个波次”实现梯次跃升。以增强公共财力为重点促进县域经济提升实力。突出财源培植、结构调整、体制改革、财税监督等四个重点，积极培育新的财源增长点；严格依法治税，规范税收秩序；集中财力保运转、保支农、保教育、保稳定、办实事；积极稳妥推进财政国库集中支付制度，加强财政管理和审计监督，提高财政运行效率和使用效益；用足用好中央“三奖一补”政策，建立财政激励机制，支持培育财源，加强资金调度，缓解县乡财政困难。坚持用规划引领城镇发展。加快九龙江沿线和沿海城镇密集区建设，改善城镇投资、就业和居住环境；引导产业和人口向县城和重点、卫星城镇集中，加快城镇群体发展步伐。

（六）致力统筹注重协调，全面发展社会事业

加快发展科教和人才事业。深化市校合作，构筑科研协作平台；培植一批自主创新优势企业，支持企业建立技术研究与开发机构；实施国家、省重点科技专项30项，实现高新技术产业产值160亿元以上。围绕“强基固本、注重质量、培育人才、保障发展”,加强发展教育事业。突出抓好农村基础教育，推进“双高普九”工作，稳步调整农村小学布局，加强实施中小学危房改造；继续扩大高中办学规模，高中阶段教育入学率达70%以上；重点发展职业教育，积极发展幼儿教育、成人教育，兴办特殊教育学校，办好老年大学，继续支持漳州职业大学、医学护理高专和漳州师范、厦大漳州校区等院校发展高等教育；健全以财政为主导的教育投入体制，积极支持社会力量办学。深入实施人才强市战略，认真落实和完善人才政策，逐步建立留住、吸引和用好人才的长效机制。

加强人口、资源和环境保护工作。完善人口计生工作新机制，切实解决计生管理不到位的突出问题，继续稳定低生育水平，提高出生人口素质。加强土地管理，落实耕地保护制度，实行单位面积投资标准管理，鼓励建设多层厂房，促进土地集约、合理、高效和可持续利用。全面整顿和规范矿产资源开发秩序。加快“两江两溪”流域水污染和生态环境综合治理，推行节水、节能、节材、节地措施，促进经济社会可持续发展。

推进文化和卫生体育事业发展。以闽南文化为主题扩展漳台文化交流合作，保护、发掘具有漳州特色的地方文化；加强创作编导人才队伍建设，鼓励文化创作上层次、出精品；启用漳州博物馆。加强高致病性禽流感、非典、艾滋病、霍乱等重大传染性疾病的防控，提高应对各类突发公共卫生事件的能力。加快城乡医疗卫生体制改革，规范社区卫生服务，引导农民积极参加新型农村合作医疗。广泛开展全民健身活动，积极备战省十三届运动会，举办市五届农运会，推进华阳体育馆、田径体育场等城乡体育设施建设。大力发展妇女儿童事业，做好老龄工作，推进残疾人事业发展。继续重视和做好档案、保密、方志等工作。

（七）努力构建和谐社会，保障群众安居乐业

加强精神文明建设。大力开展职业道德、社会公德和家庭美德教育，高度重视未成年人思想道德教育；深入开展群众性精神文明创建活动，不断提高创建成效和水平；加强国防教育、国防动员、人民防空和民兵预备役建设；深入开展“支前双拥”活动，密切军政军民关系，力争我市在新一轮创建双拥模范城（县）活动中有新的突破，全市争创双拥模范城“四连冠”。

深化平安漳州建设。认真贯彻中央《关于深入开展平安建设的意见》，大力推进“平安漳州”创建活动，积极构建社会治安综合防范体系，加强社会治安综合治理，保持严打高压态势，严厉打击各种违法犯罪活动，维护社会稳定。进一步整顿和规范市场经济秩序，加强市场监管，建立消费者权益保障机制，加快社会信用体系建设。健全社会纠纷调处机制和社会利益协调机制，认真做好群众来信来访工作，将矛盾化解在基层，消除在萌芽状态。加强防震减灾“三大工作体系”建设，完善重大突发事件处置预案，提高应急处置能力。落实安全生产责任制，强化企业主体意识，强化依法监管，强化责任管理，深入开展专项整治，坚决遏制重特大事故的发生。

重视民主法制建设。认真执行人民代表大会及其常委会的决议、决定，定期报告工作，自觉接受法律监督。积极支持政协参政议政，接受民主监督。完善政府配合人大的专题视察、审议制度，完善政府与政协的联席会议、对口协商制度。虚心听取各民主党派、工商联、无党派人士和人民团体的意见，广泛集中民意民智，推进决策科学化、民主化。进一步加强基层组织建设和民主建设。全面贯彻党的民族宗教政策，积极引导民族宗教工作与构建和谐社会相适应。

四、建设人民满意、高效运转的政府

面对发展机遇期和矛盾凸显期，我们要不断创新发展理念、发展机制、发展能力和发展模式，努力提高驾驭经济社会发展的能力，以实实在在的发展成果惠及城乡群众，努力建设人民群众满意的政府。

乘势前进，奋发有为。各级政府要把提高发展经济的能力作为执政能力建设的第一目标，抓紧机遇，致力发展，乘势而上，加快发展。要善于把握市场经济的内在要求和运行特点，遵循规律，顺势而进；要善于把握经济形势的发展变化，正确应对，趋利避害，创新机制，破解难题，以经济社会发展的新成果来体现执政能力建设的新成效。要善于把握发展大局中的自我责任，找准位置，积极作为，进一步树立和强化“重在提请、主动服务”的观念，着力改善优化漳州投资环境和发展环境。

践行宗旨，执政为民。要坚持把时时、处处、事事为群众着想作为我们工作的出发点、着力点和落脚点。对就业、医疗、社保、拖欠农民工工资等群众普遍关心的问题，要主动解决，妥善解决，及时解决。继续办好为民办实事项目。做好就业和再就业工作，力争全年新增就业5万人。进一步建立健全以养老、失业、医疗保险为主要内容的社会保障制度，适当提升城镇居民低保标准。继续抓好农村低保工作，全面落实农村五保供养政策。改进和完善征地补偿办法，逐步建立失地农民的基本生活保障制度。落实教育“两免一补”政策，全面免收农村义务教育学杂费，实行农村贫困家庭学生寄宿补助。积极探索解决群众看病难、看病贵的具体措施。加大力度清理工程欠款和拖欠农民工工资。加大扶贫开发力度，在资金、项目上扶持老区和少数民族地区加快发展。

转变职能，提高效能。继续深化行政审批制度改革，减少审批事项，规范审批行为。加强机关效能建设，严格实行绩效考评制度，提高行政效率。积极创新政府管理方式，整合、优化政务信息平台。大力精简会议、文件和各类评比活动，坚决克服形式主义，集中精力抓发展、办实事。进一步强化抓落实的主体意识，坚持和完善决策督查、项目检查等促进工作落实的有效制度，不断强化求真务实的良好氛围。

依法行政，廉洁公正。认真贯彻落实《公务员法》和《全面推进依法行政实施纲要》，加快建立权责明确、行为规范的行政执法、行政管理体制。加大行政管理的监督力度，依法规范行政行为。全面推行政务公开，进一步完善听证、公示、新闻发布、人民建议征集制度，主动接受人民群众和新闻舆论的监督，提高政务透明度和公众参与度。认真贯彻《建立健全教育、制度、监督并重的惩治和预防腐败体系实施纲要》，全面落实党风廉政建设责任制，切实纠正损害群众利益的不正之风。模范遵守廉洁从政的各项规定。加强廉洁自律教育，严肃查处违纪违法案件，从源头防止腐败现象发生。带好班子和队伍，管好亲属和身边工作人员，树立政府机关廉洁勤政的良好形象。

各位代表，漳州经济社会发展迈上了一个新的征程，我们的步伐坚实有力，我们的前景光明辉煌。让我们紧密团结在以胡锦涛为总书记的党中央周围，高举邓小平理论和“三个代表”重要思想伟大旗帜，紧紧依靠全市人民，上下一心，奋力拼搏，求真务实，开拓创新，为全面完成“十一五”和今年各项任务，为全面推进生态工贸港口城市建设而努力奋斗！
